# Volatile compositions and glandular trichomes of *Zataria multiflora* in different phenological stages under normal and drought stress conditions

**DOI:** 10.1186/s12870-024-05196-0

**Published:** 2024-05-31

**Authors:** Shahrbanoo Abbasi, Sadollah Houshmand, Tayebeh Ahmadi

**Affiliations:** 1 Department of Plant Breeding and Biotechnology, Faculty of Agriculture, Shahrekord University, P.O. Box 115, Shahrekord, Iran; 2 Department of Medical Laboratory Science, College of Science, Knowledge University, Kirkuk Road, Erbil, 44001 Iraq

**Keywords:** *Zataria multiflora*, Headspace, Glandular and non-glandular trichomes, SEM, GC-FID-MS, Drought stress

## Abstract

**Background:**

*Zataria multiflora* Boiss. is a medicinal and aromatic plant from the Lamiaceae family. It is extensively used in Iranian traditional medicine, mostly as a replacement for Thyme species. This study was focused on the analysis of chemical composition and the distribution and types of trichomes of *Z. multiflora* grown under different conditions. Equilibrium headspace analysis in combination with GC-FID-MS was used to identify volatile compounds released by aerial parts of *Z. multiflora* in development stages of 50 and 100% flowering under normal and drought-stress conditions.

**Results:**

The main constituents were p-cymene (20.06–27.40%), γ-terpinene (12.44–16.93%), and α-pinene (6.91–16.58%) and thymol (8.52–9.99%). The highest content of p-cymene (27.40%) and thymol (9.99%) was observed in the 50% flowering stage at the 90% field capacity, while the maximum γ-terpinene (16.93%) content was recorded in the 100% flowering stage under normal conditions. Using the SEM method, it was found that peltate glandular and non-glandular trichomes are distributed on the surface of the leaf, stem, and outer side of the calyx. However, capitate trichomes only are detected on the stem and calyx in the 100% flowering and beginning of blooming stages, respectively. The type and structure of trichomes do not vary in different development stages, but they differ in density. The highest number of leaf peltate glandular trichomes was observed in the vegetative and beginning of blooming stages at 50% and 90% field capacity, respectively. Non-glandular trichomes of the stem were observed with high density in both normal and stress conditions, which are more densely in 90% field capacity.

**Conclusions:**

Since this plant has strong potential to be used in the food and pharmacological industries, this study provides valuable information for its cultivation and harvesting at specific phenological stages, depending on desired compounds and their concentrations.

## Introduction

*Zataria multiflora* Boiss. is a medicinal plant of the Lamiaceae family that genus name of the plant derived from the Arabic word “Zaatar and is related to the thyme species in terms of botanical features and composition of essential oil [1, 2]. This plant is known from ancient times and geographically grown only in Iran, Pakistan, and Afghanistan [3–5]. In Iran, this plant is known by the local name Avishan Shirazi and is usually used as an anesthetic, antiseptic, and antispasmodic [3]. Aromatic compounds medicinal plants are used for perfume production, pharmaceuticals, cosmetics, food flavoring, spices, and natural food maintainers for aromatherapy and related pharmacological actions [6, 7]. Due to the increased demand for thyme, improving its cultivars under adverse environmental conditions such as drought stress will increase its natural maintenance [8, 9].

The extraction of essential oils can be used in various methods, such as hydro-distillation, steam distillation, simultaneous distillation-extraction, and headspace solid-phase microextraction [10]. Headspace gas chromatography is a technique in which the liquid or solid sample is placed in a closed vessel in a sealed vial to ensure the volatile constituents reach a balance between the sample and gas volume [11]. This technique is widely used to identify the volatile compounds in particular aromatic and medicinal plants [12]. The most common extraction technique used to obtain essential oil from aromatic plants is conventional hydrodistillation [13]. In the headspace method, volatile compounds from solid or liquid matter are separated by gas chromatography and it was used preferably for samples that cannot be injected into the machine, including fresh or dried tissue of plants, in cases where the sample amount is low [14]. Headspace sampling as the fastest and cleanest method for analyzing volatile organic compounds is generally known as a vapor-phase extraction, including the partitioning of analytics between a non-volatile liquid or solid phase and the vapor phase above the liquid or solid [15]. The headspace analysis technique provides values comparable to those of traditional volatile compounds extraction methods [16].

Although secondary metabolites do not directly play a role in primary metabolic processes, such as growth and development, they are essential for plant adaptation to a wide range of environmental stresses [17–19]. The essential oil has very complex chemical compositions. Its composition varies depending on the season, plant species, and growing conditions [7, 20, 21]. Studies have suggested that the major compositions of the essential oil of *Z. multiflora* are phenolic constituents such as thymol and carvacrol [4, 22]. The essential oil of *Z. multiflora* has the similarity of the compounds of this species with different species of thyme which the phenotypic monoterpenes, including thymol and carvacrol, which are the essential main compounds of this plant [23, 24]. There is a lack of information on the effect of drought stress conditions on EO composition in *Z. multiflora* species. Karimi and Meiners (2021) reported that the main volatile compounds of carvacrol chemotype plants did not change under drought stress. Also, in the linalool chemotype, the relative contents of thymol, *γ*-terpinene, *p*-cymene, and 1,8-octanediol were not significant to drought stress alone but decreased in response to heat stress and combined drought and heat stress [25]. The *Z. multiflora* plants collected from different habitats in Iran showed that the presence of thymol, carvacrol, and linalool chemotypes could be related to different environmental factors [26].

Trichomes, one of the epidermal structures of medicinal plants, can be divided into two types glandular trichomes and non-glandular trichomes [27]. Measure the type and number of glandular and of non-glandular trichomes is one of the techniques used for distinguishing the plants in the Lamiaceae family. The glandular trichomes responsible for the essential oil secretion exist on aerial parts of medicinal plants [28]. Seasonal changes and environmental conditions affect the growth and development of glandular trichomes, leading to differences in the morphology, types, and density inclusions of glandular trichomes. These differences may be related to environmental stress and the adaptive survival of plants [29]. The Scanning Electron Microscope (SEM) is utilized not only in chemical and physical sciences and materials but also in diverse fields such as biology and medical sciences. The high spatial resolution of SEM makes it one of the most powerful and versatile instruments available for the examination, and analysis of a wide range of the microstructural characteristics of specimens at the nanometer to micrometer length scale. A focused electron probe is used in the SEM to extract chemical and structural information point-by-point from a region of interest in the sample. Each sample for SEM must be completely dry and free from any organic contamination [30]. Yousefzadeh et al. (2022) reported that most trichomes of *T. armeniacus* were generally situated on the abaxial leaf side as compared to the adaxial one cultivated under water deficit. Also, the water deficit increased trichome density in either leaf side of *T. armeniacus* and *T. kotschyanus* species [31]. In an anatomical study, Najafpour and Alahverdi (2019) reported the high density of trichomes on the leaf adaxial surface. As well as they detected glandular trichomes of peltate and capitate on the surface of the leaf [2]. The effects of water deficit on glandular trichome density and biomass production [32] may affect the chemical compounds of essential oils [33].

The objectives current study are: (i): using headspace analysis in combination with gas chromatography/mass spectroscopy to identify volatile constituents of *Z. multiflora* in the two development stages of 50% and 100% flowering at 90 and 50% field capacity (ii): lack of data about the size and density of types of trichomes in *Z. multiflora* at different development stages, as a result, to study the effect of water stress on the physical properties of both glandular and non-glandular trichomes of *Z. multiflora* using Scanning Electron Microscopy (SEM).

## Materials and methods

*Z. multiflora* Boiss. cutting plant were obtained from fields in Abadeh city of Fars province at Iran country by Sadollah Houshmand (voucher no. 4850, with geographical coordinates 31°10’48’’N 52°40’12’’E). Cuttings of plants were planted in pots and transferred to the greenhouse in a randomized complete design with three replications. Then, to propagate the plants, cuttings were taken from the mother plant. After rooting of cuttings, these were transferred to pots measuring 25 × 30 containing a combination of peat moss, sand, and coco peat 4:2:1 ratio. Two irrigation regimes, 90% (normal) and 50% (drought stress) field capacity were used in this study, in which drought stress was applied after complete plant establishment. Finally, plant samples were harvested at 50 and 100% flowering stages in both irrigation regimes for the desired analysis.

### Gas chromatography–flame ionization detector/mass spectrometer (GC-FID-MS) and GC-MS headspace analysis

Two grams of aerial part of fresh plant material was carried out by a headspace method. Samples were introduced to 20 mL headspace vials and sealed by silicone septa. The headspace equilibrium temperature of the incubation oven and syringe was set at 100 and 105 °C, respectively, and each sample vial was placed in the heating cabinet for 30 min while being agitated. A 250 µL of headspace gas was injected into the gas chromatograph.

Gas chromatography (GC) was carried out using an Agilent 5975 C device equipped with an FID detector and HP-5MS capillary column (30 m × 0.25 mm I.D., 0.25 μm film thickness). The Oven temperature was programmed for 70–200 °C, at a rate of 5 °C/min, and held for 10 min at 290 °C. The carrier gas was helium (flow rate: 1.1 mL/min). The injection temperature was set at 290 °C.

Gas chromatography-mass spectrometry (GC-MS) analysis was performed using an Agilent 5975 C-7890 instrument that was equipped with an HP-5MS capillary column (30 × 0.25 mm ID film thickness × 0.25 mm) at the Islamic Azad University of Isfahan (Khorasgan). Carrier gas was helium with a flow rate of 1.1 mL/min, and the temperature program was set as follows: initial temperature 60 °C; then increased from 60 to 250 °C at a rate of 5 °C/min and held for 10 min at 290 °C. The inlet pressure was 9.43 psi. Injector, detector, and ion source temperatures were set at 290 °C; 280 °C and 230 °C temperatures were set at 280 and 150 °C, respectively. Ionization of the sample ingredients was carried out in the EI mode (70 eV) with a scan range and time of 20–555 amu and 1.60 s, respectively.

RI (linear retention indices) for all constituents was identified by injection of the hexane solution, including the homologous series of C8-C26 *n*-alkanes [34]. The identification of the volatile compositions was accomplished by the visual interpretation, comparing their retention indices and mass spectra with literature data [35], by computer library search (HP Chemstation computer library NBS75K.L, NIST/EPA/NIH Mass Spectral Library 2.0 and Willey275.L). Constituent concentrations (as % content) were evaluated by integrating their corresponding chromatographic peak areas from GC-FID analysis.

### Scanning electron microscopy (SEM)

Leaf, stem, and calyx samples of the *Z. multiflora* were collected in the different development stages (50% and 100% flowering). To obtain comparable results, the leaves and stems of three nodes from the plant apex were used for SEM analyses at the SEM Laboratory of Isfahan University of Technology [36]. Rapid and suitable fixation method is first and most important step after sample selection for the study of tissues. The samples were placed in 4% glutaraldehyde for 6 h (room temperature) and then in 0.01 M phosphate buffer (pH 7.0, 4 °C) for 48 h. The samples were dehydrated in an ethanol series at concentrations of 15, 30, 50, 70, 90, 96, and 99.8% and twice in absolute ethanol for 15 min. When slices dehydrated, they were transferred to acetone. In this time, samples were dried by freeze dryer VaCo5 (ZiRBUS). Then, all specimens were coated with a thin layer of gold [37, 38] that to prevent electrical charging is essential a uniform coat in SEM. Eventually, the leaves, stem and calyx surfaces were investigated and imaged under were made to determine the density and types of the trichomes using a Philips XI30 scanning electron microscope an acceleration voltage of 20 kV.

### Statistical analysis

Hierarchical cluster analysis (HCA) was carried out based on the ward’s method with arithmetic average (UPGMA dendrogram) to classify important components of *Z. multiflora* in the two phenological stages at 90% and 50% field capacity by SPSS (SPSS Inc. Chicago IL.V. 16.0) software. Principal component analysis (PCA) was employed to visualize the similarities or differences in the proportion of some components under normal and drought-stress conditions using the StatGraphics (version 18.01.6) software, and the results are presented as bi-plots. The correlation coefficients between the components were evaluated using Pearson’s correlation coefficient by SAS (version 9) software.

## Results

### Volatiles components of* Z.multiflora*and statistical analysis

GC-FID and GC-MS analyses of the headspace volatiles components released from fresh *Z. multiflora* revealed 28 compounds (Table 1), representing 95.03–97.55% of the total. The major components of the headspace of *Z. multiflora* were p-cymene (20.06–27.40%), γ-terpinene (12.44–16.93%), and α-pinene (6.91–16.58%). The most abundant class of compounds was monoterpene hydrocarbons (66.33–81.92%). Similarly, Abkenar et al. (2008) identified thymol, carvacrol, p-cymene, Linalool, caryophyllene, and α-terpineol as the majors of the headspace of dry plant material of *Z. multiflora* [39].

**Table 1:** Volatile components of Z. *multiflora* in two different development stages under normal (N) and drought stress (D) conditions

		50% Flowering		100% Flowering	
RI	Compound	*N*	D	*N*	D
928	α-Thujene	6.85	4.55	6.79	3.88
938	α-Pinene	6.91	16.58	10.24	8.33
956	Camphene	0.45	0.71	0.66	0.46
978	Sabinene	0.16	0.20	0.29	
985	β-Pinene		1.82	1.31	1.03
992	Myrcene	10.40	6.41	10.46	8.13
1015	α-Phellandrene		0.88		0.90
1025	α-Terpinene	7.97	5.89	8.26	5.92
1038	*p*-Cymene	27.40	20.06	26.59	24.78
1052	*E*-Ocimene	0.27	0.20		0.21
1068	γ-Terpinene	16.20	13.38	16.93	12.44
1096	Terpinolene	0.35	0.26	0.39	0.25
1117	Linalool	2.07	0.98	1.79	1.50
1206	Terpinen-4-ol	0.81	0.51	0.56	0.52
1224	α-Terpineol		0.33	0.30	
1242	Thymol methyl ether	0.58	0.15	0.42	0.81
1328	Thymol	9.99	5.06	8.52	6.18
1443	β-Caryophyllene	3.06	2.89	2.51	2.36
1455	Aromadendrene	0.69	0.26	0.26	
1461	α-Selinene			0.45	
1462	γ-Gurjunene				0.59
1513	α-Farnesene			0.36	0.34
1519	*(Z)*-Nerolidol	0.87			
1978	(*E*)-9-Octadecenal		15.69		
1798	1-Eicosanol				2.67
2194	1-Hexacosanol				16.25
	Monoterpene hydrocarbons	76.96	70.94	81.92	66.33
	Oxygenated monoterpenes	13.45	7.03	11.59	9.01
	Sesquiterpene hydrocarbons	3.75	3.15	3.08	3.29
	Oxygenated sesquiterpenes	0.87			
	Alkanes		15.69		18.92
	Total identified	95.03	96.81	96.59	97.55

*RI* Retention indices on the HP-5MS column

As the majority of the Lamiaceae species, *Z. multiflora* also shows chemical polymorphism. The essential oil composition of this species depends on genetic and environmental factors. Therefore, there are four chemotypes of *Z. multiflora* found in Iran, i.e., thymol (6.0-54.9%), carvacrol (0.7–50.6%), Linalool (1.2–46.8%), and p-cymene (1.6–14.8%) [40], Moreover, Mousavi et al. (2008) reported carvacrol (71.20%), γ-terpinene (7.34%), and α-pinene (4.26%) as main compounds of essential oil, while Mahboubi et al. (2010) identified thymol (38.70%), carvacrol (15.30%) and p-cymene (10.20%) as the most abundant [41, 42]. Moreover, according to the results presented in Table 1, stress conditions decreased the content of the major compounds in both phenological stages of *Z. multiflora*, including thymol, p-cymene, and γ-terpinene.

**Fig. 1:**
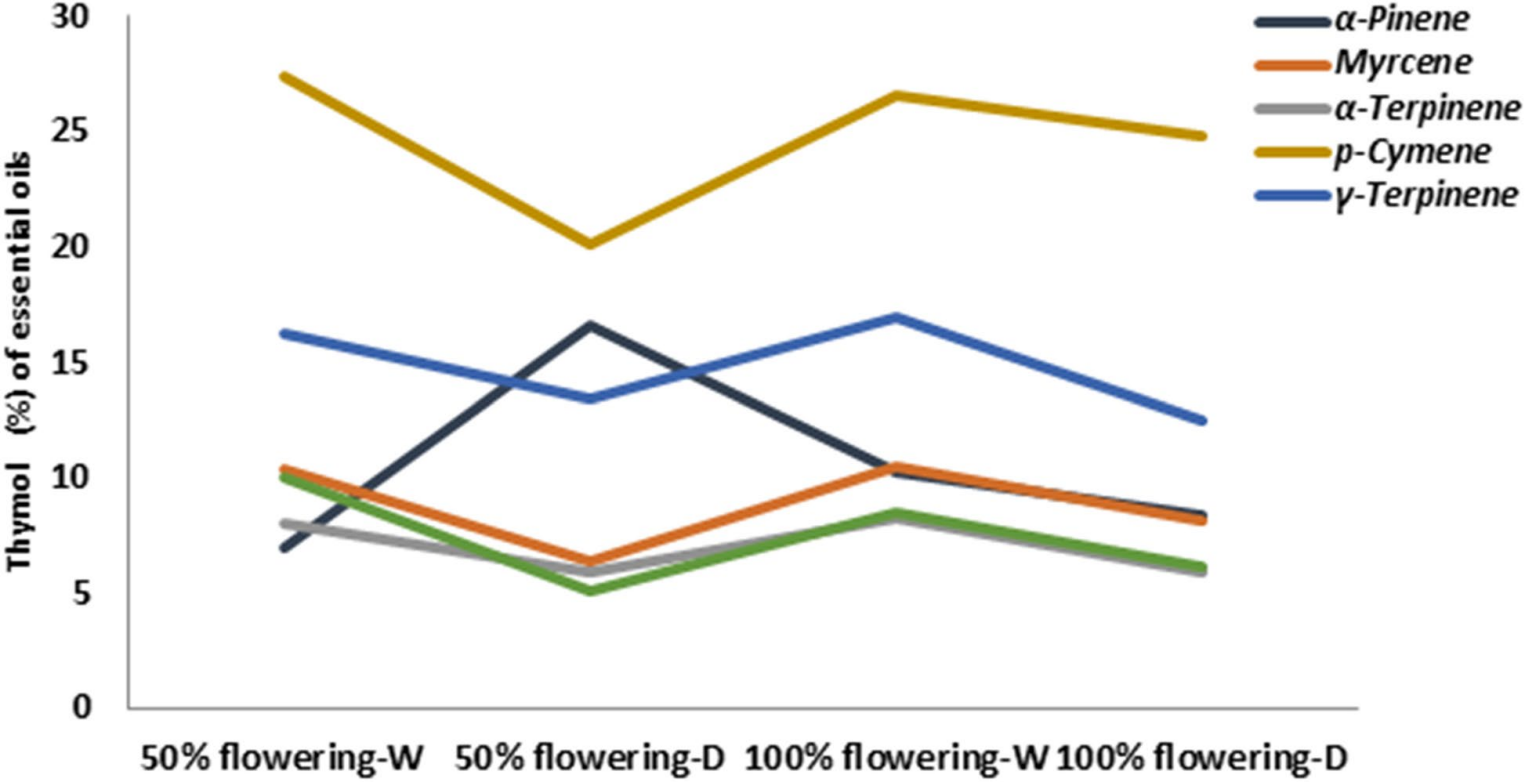
Amounts of thymol, α-pinene, p-cymene, γ-terpinene, α-terpinene and myrcene components in *Z. multiflora* at 50 and 100% flowering under normal (N) and drought stress (D) conditions

Chemical components including thymol, α-pinene, p-cymene, γ-terpinene, α-terpinene, and myrcene contain more than 65% of compounds of *Z. multiflora* in two different development stages under normal (N) and drought stress (D) conditions (Fig. 1). Therefore, an analysis was carried out between these compounds at 50 and 100% flowering stages. As shown in Table 1, the chemical compounds of p-cymene and γ-terpinene showed a significant difference in the 50% and 100% flowering stages at 90% field capacity than under normal which compounds both had the highest amount in the 50% flowering at stage 90% field capacity. Thymol has a significant difference in the 50% flowering stage at 90% field capacity with other conditions, which has the highest amount (9.99%) at this stage. This is the result of Table 1. There was a significant difference between the 50% and 100% flowering stages with 90% field capacity than under normal for the chemical compounds of myrcene and α-terpinene.

### Hierarchical cluster analysis (HCA) and principal component analysis (PCA)

**Fig. 2:**
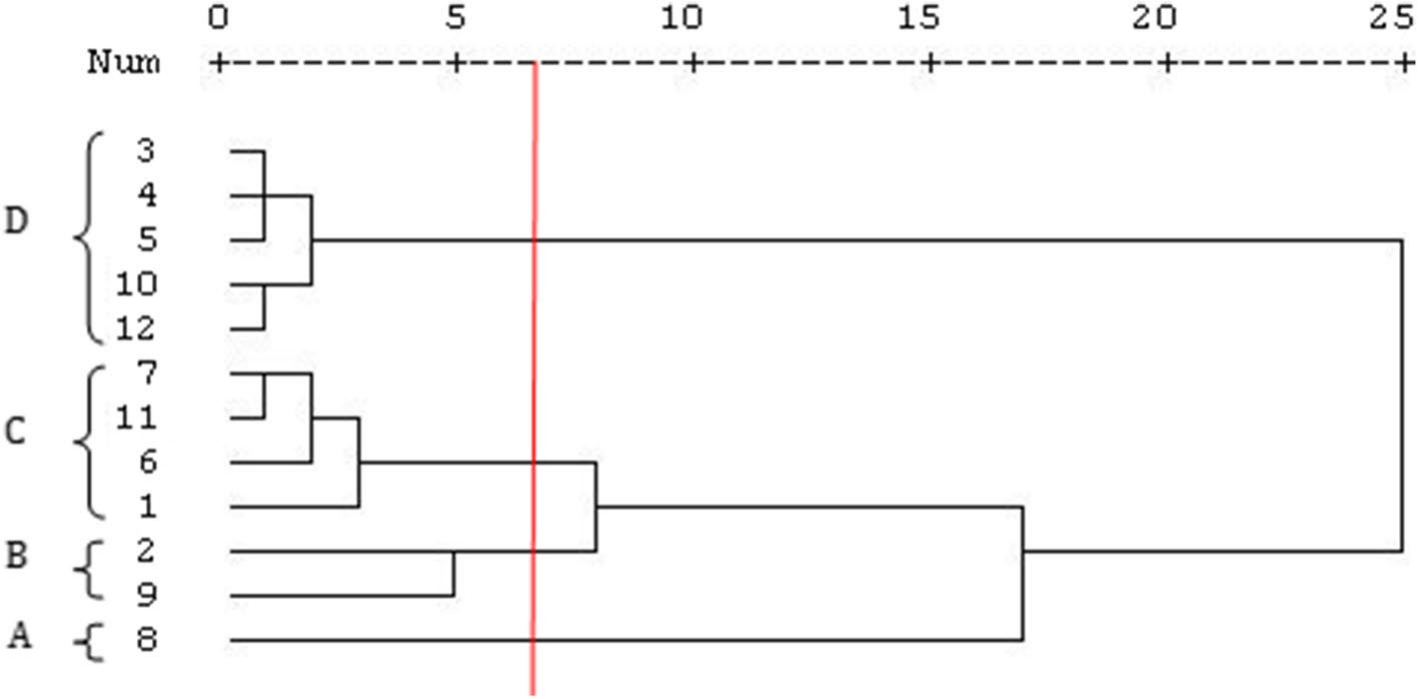
The UPGMA (with arithmetic average) dendrogram for the essential oil constituents of *Z. multiflora* species under 90 and 50% field capacity using the ward method. Numbers (1–10) represent the identified constituents of essential oil: 1. α-Thujene, 2. (R)-α-pinene, 3. Camphene, 4. Sabinene, 5. β-Pinene, 6. Myrcene, 7. α-Terpinene, 8. p-Cymene, 9. γ-Terpinene, 10. Linalool, 11. Thymol and 12. β-Caryophyllene

**Fig. 3:**
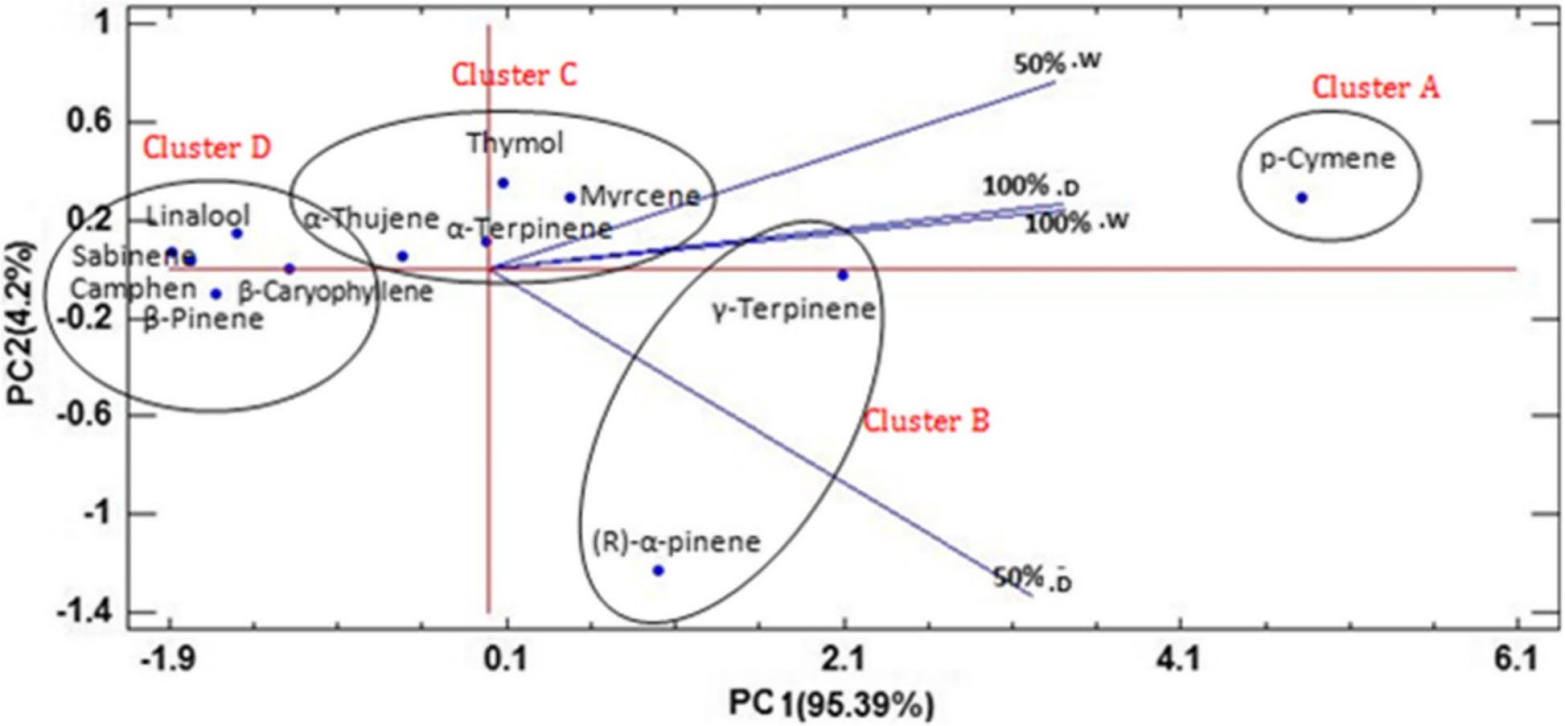
PCA analysis of essential oil components of *Z. multiflora *species under normal (N) and drought stress (D) conditions in the development stages of 50 and 100% flowering

To evaluate the similarities and relationships among some volatile components with higher frequencies in the *Z. multiflora*, a hierarchical cluster analysis was conducted based on the Euclidean distance. The HCA results are presented in the form of a dendrogram (Fig. 2). Based on this analysis, the components were categorized into four groups. The first group consisted of the p-cymene. In both phenological stages, this aromatic monoterpene had the highest values in comparison to other compounds under both normal and stress conditions, and it formed the first group alone. The α-pinene and γ-terpinene were classified in the second group. The third group comprised α-thujene, myrcene, α-terpinene, and thymol, while the main compounds of the fourth group were camphene, sabinene, β-pinene, Linalool, and β-caryophyllene. The PCA analysis of the *Z. multiflora* headspace components revealed four groups (Fig. 3). The first and second components explained 99.59% of the total variation, while the first PC1 and PC2 explained 95.39% and 4.2% of the total variation, respectively.

### Correlations between the main components

There were the highest correlation coefficients in the correlation matrix between α-terpinene and γ-terpinene with terpinolene (r_0.01_=0.99), as well as γ-terpinene and α-thujene (r_0.01_=0.98) (Table 2). Also, there was a significant positive correlation between two components of γ- terpinene and p-cymene with terpinolene (r_0.01_=0.99 and r_0.01_=0.72, respectively) and linalool (r_0.01_=0.72 and r_0.01_=0.98, respectively). A positive and significant correlation was shown between α-thujene (r_0.01_=0.85), α-terpinene (r_0.01_=0.93), p-cymene (r_0.01_=0.96), γ-terpinene (r_0.01_=0.85), terpinolene (r_0.01_=0.89), linalool (r_0.01_=0.96), and thymol (r_0.01_=0.95) with Myrcene. Moreover, negative correlations were recorded between each pair of p-Cymene and α-Pinene (r_0.01_=-0.92), Linalool and α-Pinene (r_0.01_= -0.89), and Thymol and α-Pinene (r_0.01_=-0.96), Myrcene and α-Pinene (r_0.01_=-0.77).

**Table 2:** Correlation coefficients between main constituents of the *Z. multiflora* plant at phonological stages of 50% and 100% flowering

	α-Thujene	α-Pinene	Camphene	Sabinene	Myrcene	α-Terpinene	*p*-Cymene	γ-Terpinene	Terpinolene	Linalool	Thymol	β-Caryophyllene
**α-Thujene**	1.00											
**α-Pinene**	-0.38^ns^	1.00										
**Camphene**	-0.001^ns^	0.87**	1.00									
**Sabinene**	0.69*	0.36^ns^	0.71	1.00								
**Myrcene**	0.85**	-0.77*	-0.38^ns^	0.32^ns^	1.00							
**α-Terpinene**	0.97**	-0.5^ns^	-0.08^ns^	0.63*	0.93**	1.00						
**p-Cymene**	0.68*	-0.92**	-0.61*	0.04^ns^	0.96**	0.80**	1.00					
**γ-Terpinene**	0.98**	-0.33^ns^	0.19^ns^	0.76*	0.85**	0.98**	0.66*	1.00				
**Terpinolene**	0.96**	-0.38^ns^	0.07^ns^	0.72*	0.89**	0.99**	0.72*	0.99**	1.00			
**Linalool**	0.76*	-0.89**	-0.61*	0.09^ns^	0.96**	0.83**	0.98**	0.72*	0.74*	1.00		
**Thymol**	0.89**	-0.74*	-0.45^ns^	0.3^ns^	0.95**	0.91**	0.90**	0.84**	0.83**	0.96**	1.00	
**β-Caryophyllene**	0.41^ns^	0.16^ns^	0.03^ns^	0.31^ns^	0.01^ns^	0.21^ns^	-0.09^ns^	0.30^ns^	0.15^ns^	0.11^ns^	0.32^ns^	1.00

^ns^: non-significant

^**^ Significant at (*P*<1%)

^*^ Significant at (*P*<5%)

### Types, distribution, and structure of leaf glandular trichomes

**Fig. 4:**
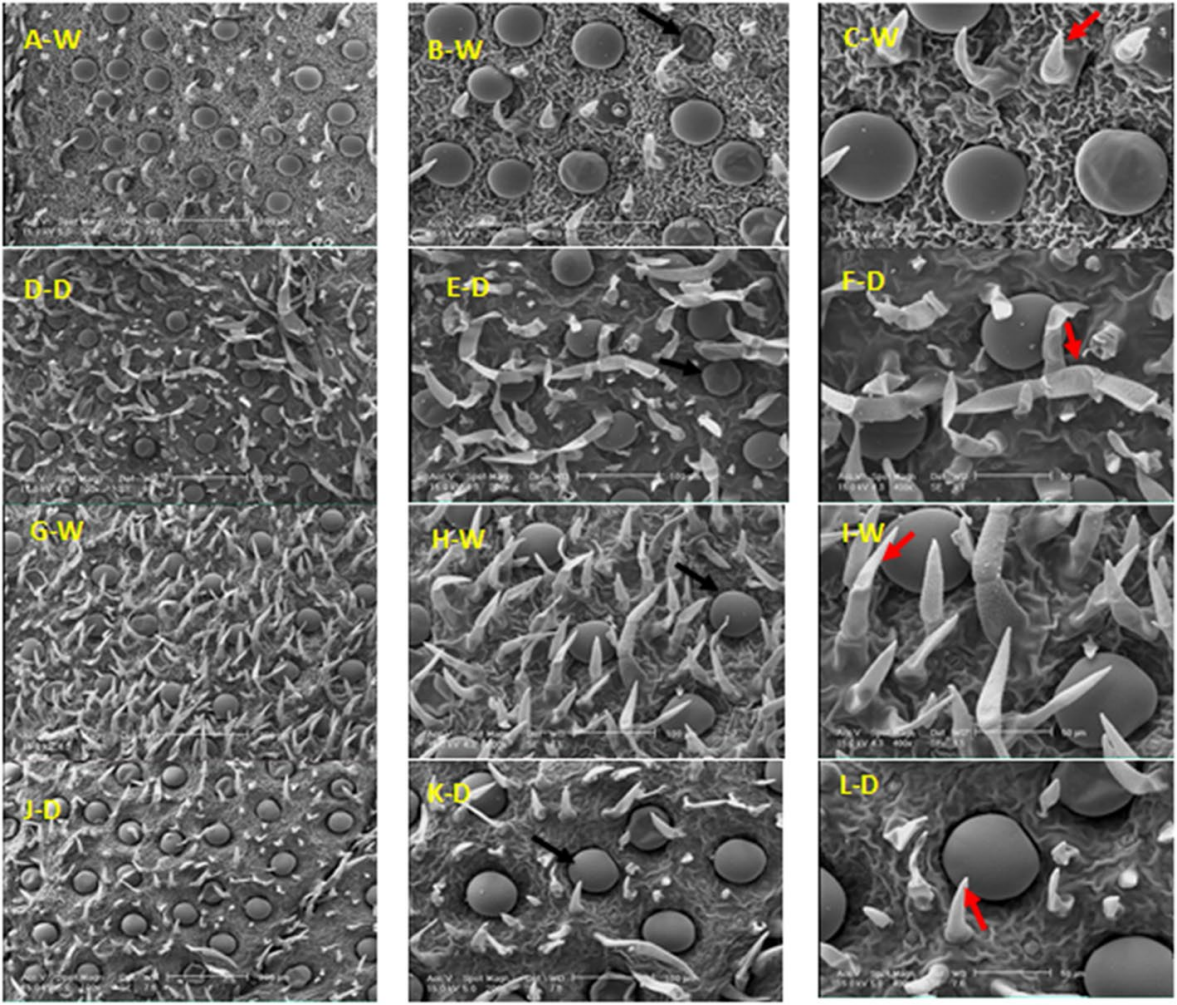
SEM micrographs showing the morphology of glandular and non-glandular trichomes on a leaf of *Z. multiflora *in the vegetative(A-F) and beginning of blooming (G-L) stages under two conditions of 90% (C) and 50% (D) field capacity. Peltate glandular (  ) and non-glandular (  ) trichomes

 Leaves of *Z. multiflora* contain peltate glandular and non-glandular trichomes (Fig. 4). It was found that the glandular trichomes density of peltate was higher in normal (90% field capacity) than stress (50% field capacity) conditions on the epidermis of *Z. multiflora* leaf. This type of trichomes has a smooth surface in the vegetative stage, which is probably related to the cuticle’s dependence on the secretions of the upper cell wall. They include one basal epidermal cell, a stalk cell, and a head of 12 secretory cells (Fig. 4B). In both conditions of 90 and 50% field capacity, there are short (unicellular) and elongated (multicellular) non-glandular trichomes (Fig. 4F).

Peltate glandular trichomes were identified with higher density at 50% filed capacity at the beginning of the blooming stage (Fig. 4J). Also, non-glandular trichomes were identified with higher density in normal than stress conditions, unlike the vegetative stage. Non-glandular trichomes that are unbranched, simple, and unicellular or multicellular have a warty surface. Peltate glandular trichomes are located in deep concavities at the beginning of blooming than in the vegetative stage at the 50% field capacity (Fig. 4L).

**Fig. 5:**
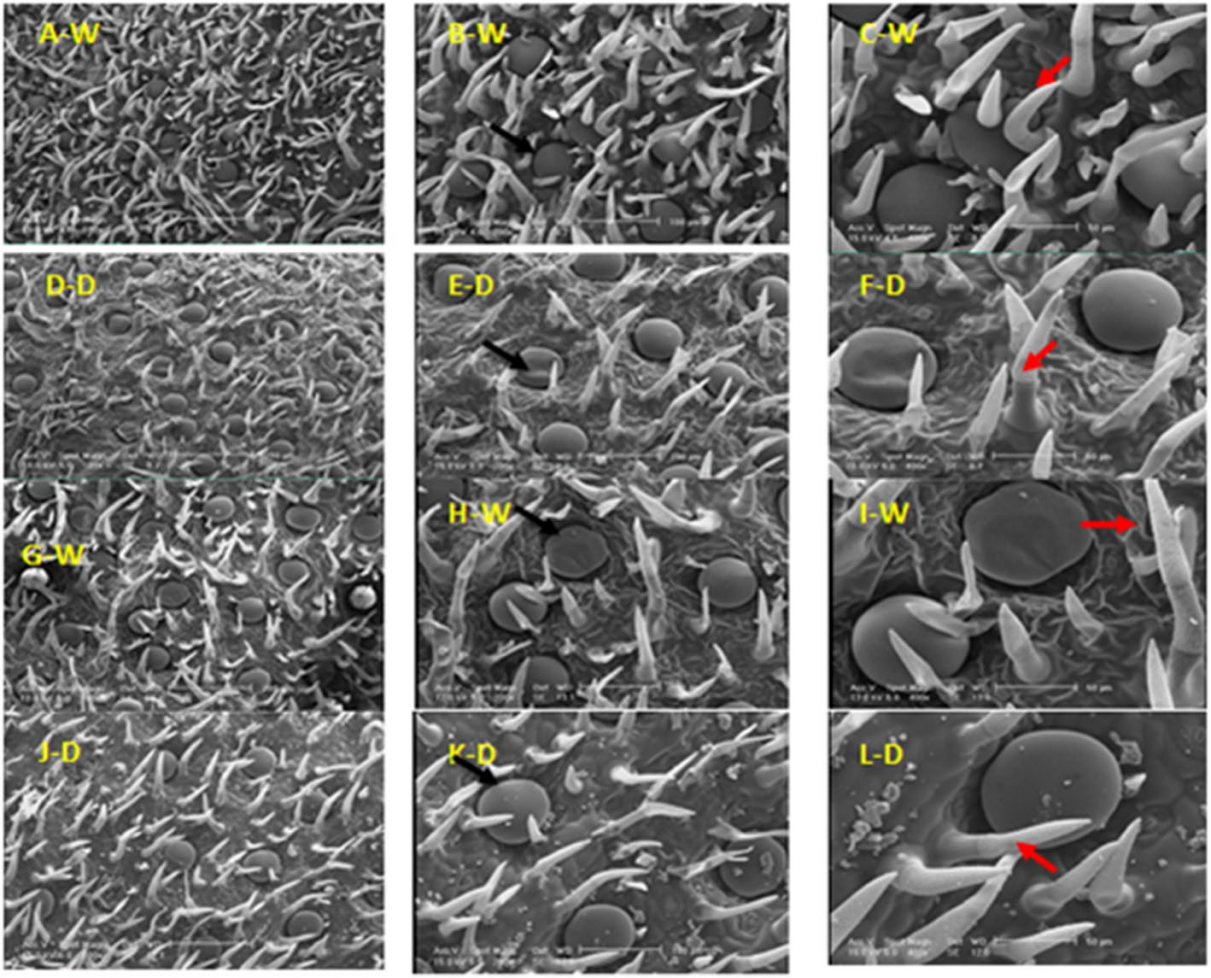
SEM micrographs showing the morphology of glandular and non-glandular trichomes on leaf of *Z. multiflora *in the 50% (A-F) and 100% (G-L) flowering stages under two conditions of 90% (N) and 50% (D) field capacity. Peltate glandular (  ) and non-glandular (  ) trichomes

 In the 50% flowering stage were observed both types of glandular and non-glandular trichomes with high density (Fig. 5). Non-glandular trichomes with a warty surface and are unbranched and unicellular to three-cellular were more frequent and numerous than peltate trichomes in the 90% field capacity (Fig. 5A). At this stage of development, such as the beginning of the blooming stage, peltate trichomes were placed in the pit (Fig. 5B-F).

In the 100% flowering stage, there are peltate glandular trichomes elongated with more density in the normal compared to the stress condition (Fig. 5G). Of course, the density of this type of trichomes has decreased compared to the previous development stages. Along with growth, some of the peltate trichomes sank gradually. Non-glandular trichomes were observed with comparatively similar densities in both normal and stress conditions which there are both non-glandular trichomes of short and elongated in both conditions.

### Types, distribution, and structure of stem glandular trichomes

**Fig. 6:**
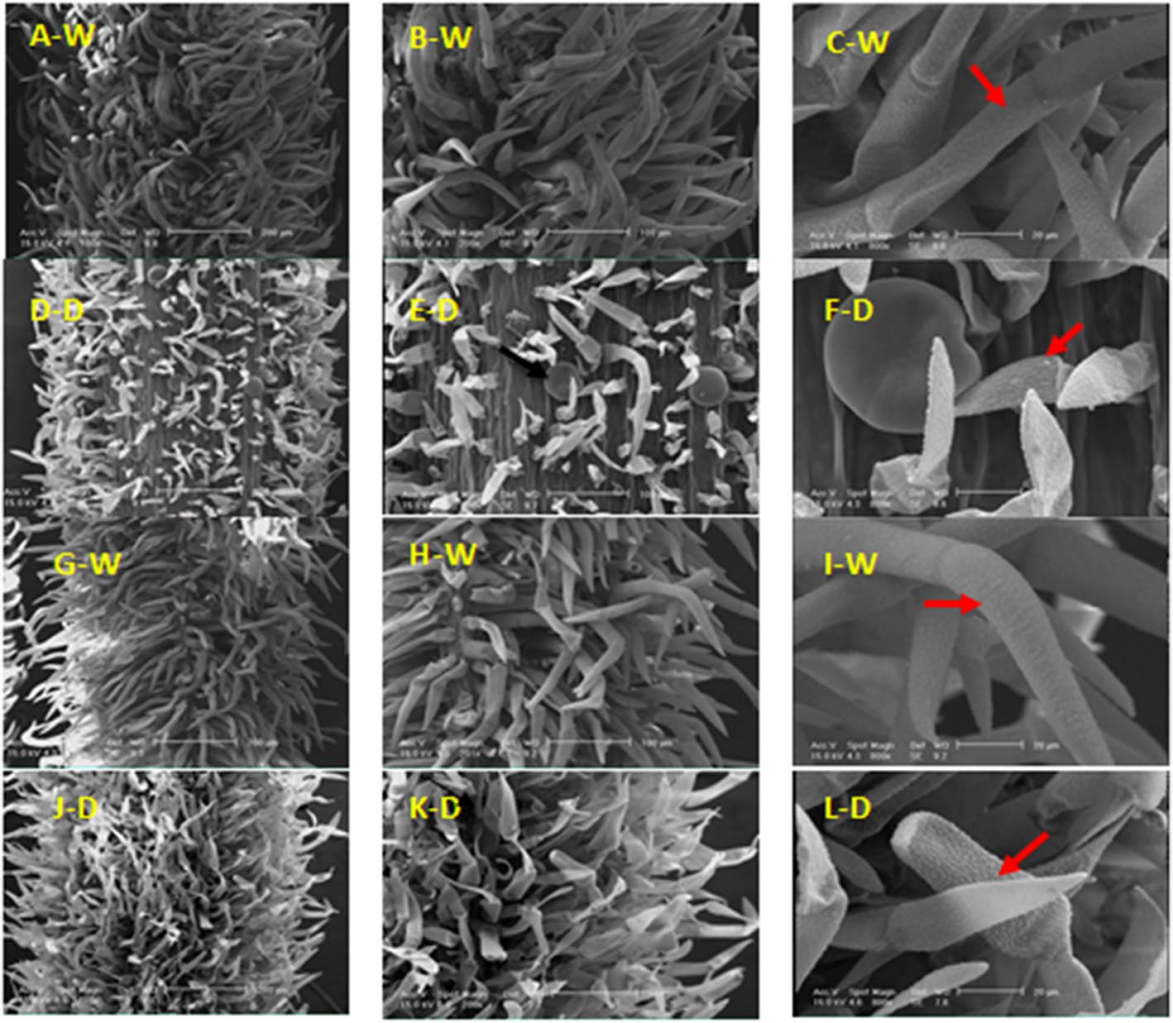
SEM micrographs showing the morphology of glandular and non-glandular trichomes on the stem of *Z. multiflora *in the vegetative (A-F) and beginning of blooming (G-L) stages under two conditions of 90% (N) and 50% (D) field capacity. Peltate glandular (  ) and non-glandular (  ) trichomes

 In the vegetative stage, peltate glandular and non-glandular trichomes were found on surfaces of the stem at 50% field capacity, whereas only peltate glandular trichomes were found at 90% field capacity (Fig. 6A-F). Non-glandular trichomes with higher density were observed in normal (90% field capacity) relative to stress (50% field capacity) conditions. In conditions of normal, non-glandular trichomes are unbranched and multicellular. However, under stress conditions, non-glandular trichomes are unicellular or bicellular types and rarely were observed as multicellular.

**Fig. 7:**
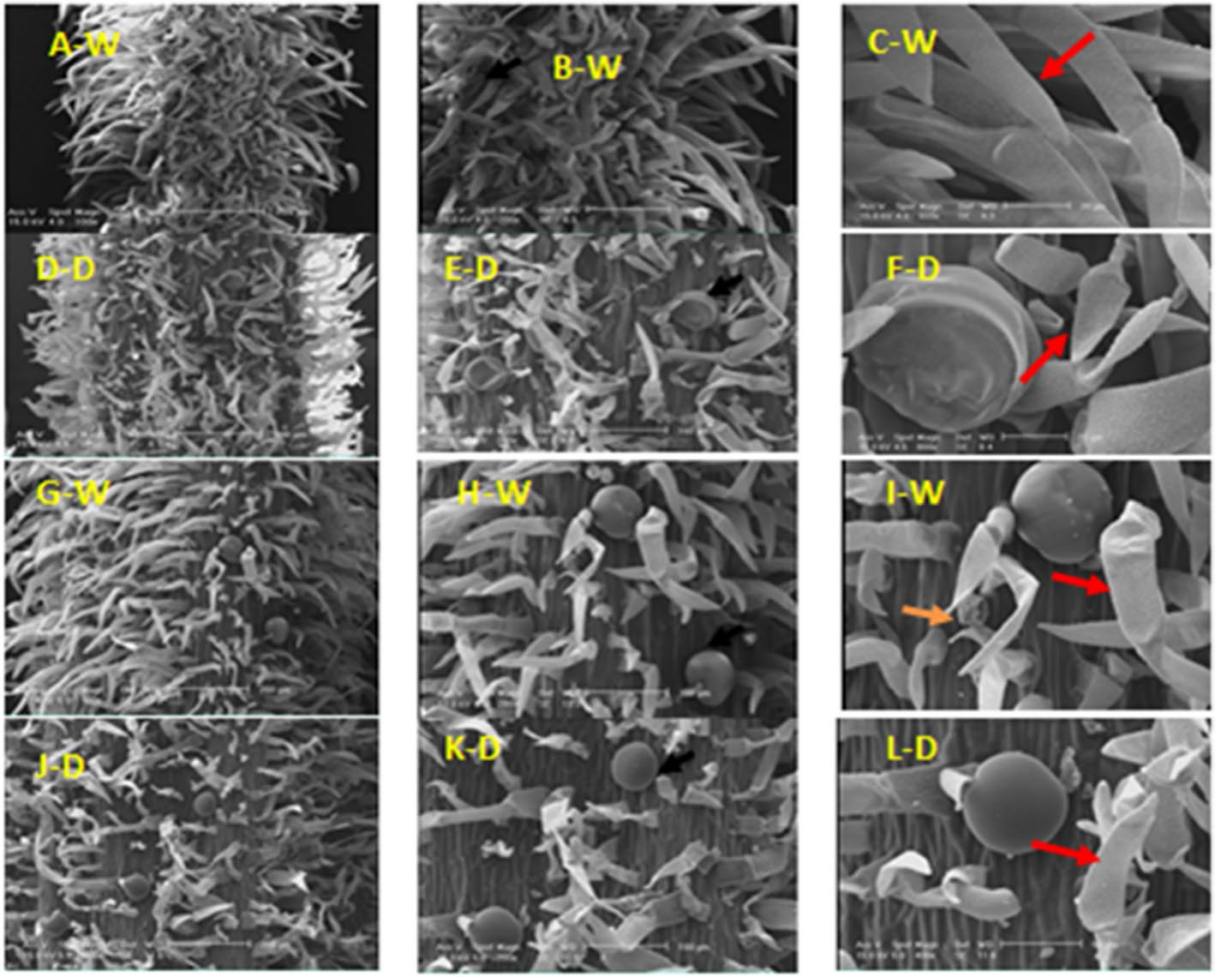
SEM micrographs showing the morphology of glandular and non-glandular trichomes on the stem of *Z. multiflora *in the 50% (A-F) and 100% (G-L) flowering stages under two conditions of 90% (N) and 50% (D) field capacity. Peltate (  ), Capitate glandular (  ) and non-glandular (  ) trichomes

 At the beginning of the blooming stage, there are non-glandular trichomes with high density on the stem in both normal and stress conditions (Fig 6G-J). Peltate glandular trichomes were not detected on the stem. In the development stage of 50% flowering, peltate glandular trichomes are present in both conditions of 90% and 50% field capacity, which is higher in terms of stress (Fig. 7A-F). The peltate trichomes have a large disc-like head and a short stalk. The head of peltate trichomes looks like that of the peltate one on leaves. Secretion of peltate trichome was released by the cuticle rupture (Fig. 7E).

Two types of glandular trichomes, including peltate and capitate, exist on the stem in both conditions of normal and stress at the 100% flowering stage (Fig.7G-J). With the release of secretion, the cuticle of the capitate glandular trichome becomes shriveled (Fig. 7H). The secretion of capitate glandular trichomes was extruded through the cuticle (Fig. 7H). Non-glandular trichomes are multicellular on the stem. The surface of non-glandular trichomes is warty in the 90 and 50% field capacity (Fig. 7I).

### Types, distribution, and structure of calyx glandular trichomes

**Fig. 8:**
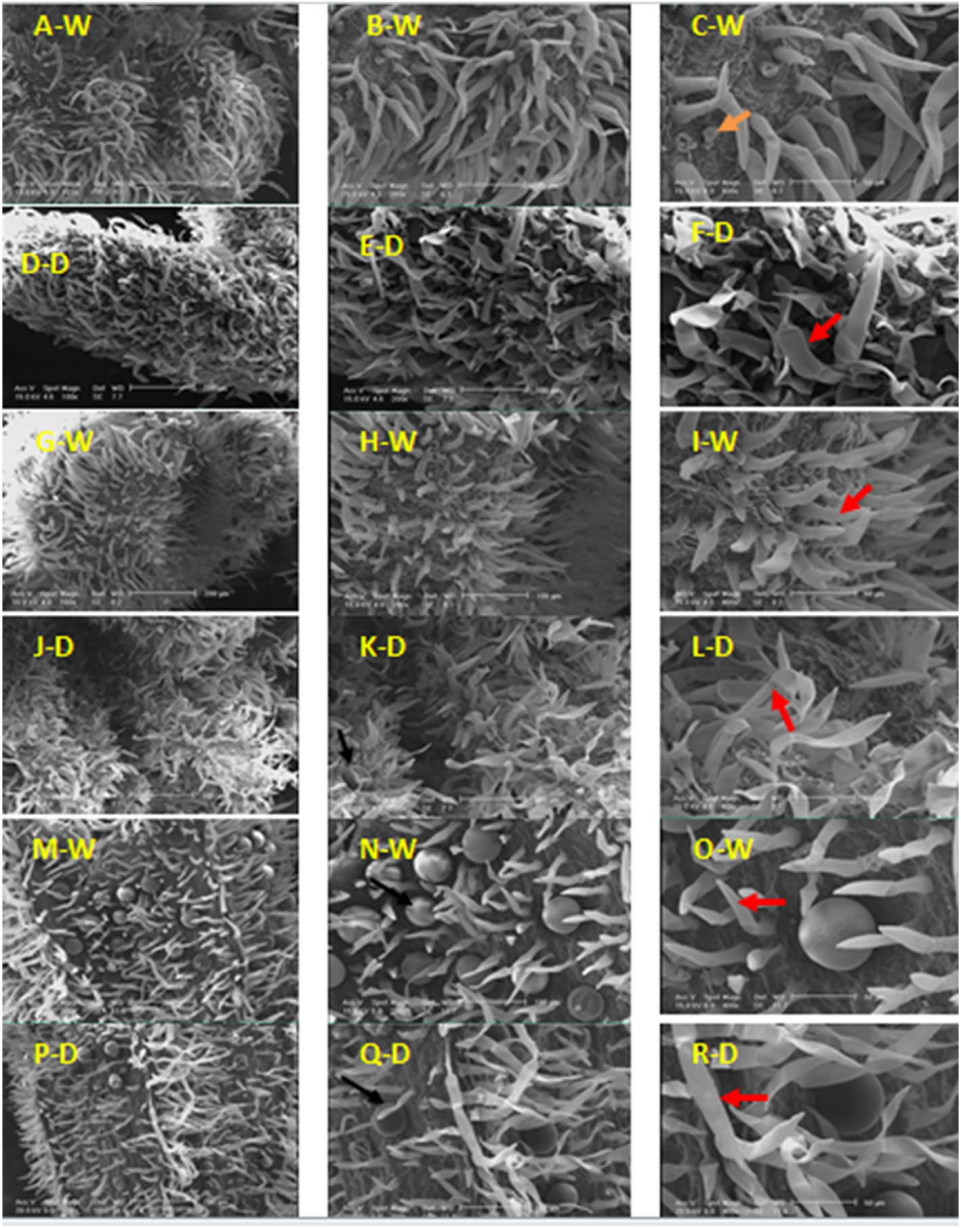
SEM micrographs showing the morphology of glandular and non-glandular trichomes on the calyx of *Z. multiflora* at the beginning of the blooming (A-F), 50% (G-L) and 100% (M-R) flowering stage at the 90% (N) and 50% (D) field capacity. Peltate glandular (  ), Capitate glandular (  ) and non-glandular (  ) trichomes

 There are peltate and capitate glandular trichomes with low density in normal conditions at the beginning of blooming (Fig. 8A-C). Only peltate glandular trichomes are distributed on the calyx surface (Fig. 8D). In normal conditions, peltate glandular trichomes have been affected by depression and evacuation of essential oil, But under stress, these types of trichomes are solid. The head of the peltate trichomes has 4 secretory cells (Fig. 8E-F).

Peltate glandular trichomes were not observed under normal (90% field capacity) conditions, peltate glandular trichomes of calyx were placed on the epidermis, not on the inside of the cavity. Non-glandular trichomes with high density have existed in both normal and stress conditions on the outer surface of the calyx in the 50% flowering stage (Fig. 8G). Two types of non-glandular trichomes were identified which are short and stretched. These trichomes are unbranched and unicellular to multicellular (Fig. 8K).

Peltate and capitate glandular trichomes were discovered higher in conditions of 90 than 50% field capacity at 100% flowering stage (Fig. 8M). At this stage, Peltate glandular trichomes were observed with a much higher density than the previous stages of growth (beginning of blooming and 50% flowering stages) in both normal and stress conditions.

## Discussion

The total amount of monoterpenes was decreased by water deficiency. The most amounts of monoterpene hydrocarbons (81.92%) and oxygenated monoterpenes (13.45%) were observed in the 100% and 50% flowering stages at 90% field capacity, respectively. Some components reduced significantly under stress, such as myrcene, α-terpinene, p-cymene, γ-terpinene, and α-thujene in both stages of 50 and 100% flowering while (R)-α-pinene was increased in the 50% flowering stage. Shafiee and Javidnia (1997) reported that the major constituents of *Z. multiflora* obtained from the Yazd Province of Iran are phenolic compounds of carvacrol (61.29%) and thymol (25.18%) [22], while in the current study, the main phenolic constituent of *Z. multiflora* is thymol (71.12%) only and we didn’t find any carvacrol. The many studies indicated the positive efficacy of drought stress on the volatile compounds of essential oil in medicinal plants [17, 42–44], some studies also proved negative effects. Akula and Ravishankar (2011) reported a decrease in secondary metabolites under environmental stress conditions [45]. Manukyan (2011) stated that the total content of terpenoids in lemon balm and sage plants was lower under drought stress [46]. Ghasemi Pirbalouti et al. (2014) also observed a significant decrease in thymol content in *T. defenses* under stress conditions [47].

Results of principal component analysis showed that the first PC1 has a positive correlation with p-cymene and thymol. The higher γ-terpinene than p-cymene amount causes more synthesis of thymol and the reason; p-cymene alone formed the first group [48]. Therefore, at the 50 and 90% flowering stages, more thymol was synthesized under normal conditions where the concentration of p-cymene is higher. Tohidi et al. (2017) also reported a positive correlation between PC1 with p-cymene and thymol in several *Thymus* species from Iran [49].

A strong correlation was found between thymol with γ- terpinene (r_0.01_=0.84) and p-cymene (r_0.01_=0.90). As well as, there was a positive correlation between γ-terpinene and p-cymene (r_0.01_=0.66). Therefore, since these two components are precursors of thymol, this finding indicates that their production is highly dependent [49].

It was determined using SEM that *Z. multiflora* has two types of glandular trichomes, including peltate and capitate. The capitate glandular trichomes only are dispersed on the stem surface in the 100% flowering stage and on the outer side of the calyx at the beginning of the blooming and 100% flowering stages. The highest number of leaf peltate glandular trichomes was observed in vegetative and beginning of blooming stages at 50% and 90% field capacity, respectively. Except for the beginning of the flowering stage, in three other stages of development, the density of these trichomes has decreased under stress conditions. The highest non-glandular trichome density was detected at the beginning of blooming and 50% flowering at 90% field capacity. The morphology and structure of glandular and non-glandular trichomes are similar in the different development stages at 90 and 50% field capacity. Talebi et al. (2018) investigated 12 species of Nepeta using SEM and identified two types of peltate and capitate glandular trichomes and non-glandular branched and unbranched trichomes [50]. Using SEM on the leaf surface of lavender was detected three types of glandular trichomes: were peltate, capitate, and non-glandular. Peltate glandular and non-glandular trichomes were located on the adaxial and abaxial surfaces of leaves and sepals. However, only capitate glandular trichomes were shown on the petals. Like the peltate glandular trichomes in other Lamiaceae plants, peltate glandular trichomes of lavender cultivars were also composed of three cell types base, stalk, and head. Peltate glandular trichomes consist of one stalk cell, one basal cell, and several multicellular heads [51].

Non-glandular trichomes of stem were observed with high density in both normal and stress conditions, which are more densely in 90% field capacity. In the two conditions of normal and stress, non-glandular trichomes were placed on both sides of the stem of the multicellular type and short (unicellular or bicellular) in the middle. The distinction between the two types of peltate and capitate glandular trichomes is also very obvious based on the differences in morphology, which is often related to the mechanism of essential oil release [52].

Generally, the structure and morphology of stem peltate glandular and non-glandular (unicellular to multicellular with warty surface) trichomes are similar in each of the four development stages studied.

Two types of glandular trichomes of peltate and capitate are very different in morphology and structure on the calyx. Trichomes of non-glandular exist on the outer side of the calyx in both conditions with high density. However, under conditions of stress, they took a thin sheet-like shape due to water shortages. Dmitruk et al. (2010) found the highest density of non-glandular and glandular trichomes on the abaxial surface of the calyx and stem [53]. Jia et al. (2013) identified two types of glandular trichomes, namely, peltate and capitate, on the external surface of the calyx using SEM [52].

In general, peltate glandular and non-glandular trichomes were observed on leaf, stem, and calyx surfaces in four stages of vegetative, beginning of blooming, 50%, and 100% flowering in the studied species. Non-glandular trichomes that are present in all organs are more abundant on the stem and calyx.

The leaves of *the Z. multiflora* plant have been used to determine the amount of volatile components and according to the results obtained the effect of drought stress and phenological stage of the plant on the density of peltate glandular trichomes was determined. By reducing the amount of water from 90 to 50% of the field capacity, the amount of the main compounds identified, including α-pinene, p-cymene, γ-terpinene and thymol, in both phenological stages of 50 and 100% flowering has been faced with a decrease. Since p-cymene is the main product consisting of α-terpene, it can be said that the reason for p-cymene being higher in normal conditions is the higher amount of α-terpinene that this monoterpene spontaneously It forms p-cymene even faster than γ-terpinene, which due to the greater density of the peltate glandular trichomes in normal conditions, the amount of both volatiles components is higher. According to the results, at the 50% flowering stage, the density of peltate glandular trichomes decreased with a reduction in water content from 90 to 50% field capacity, and as a result, more thymol content was observed in normal conditions. In the 100% flowering stage, as well as in the 50% flowering stage, trichome density was higher in normal conditions compared to stress, but compared to 50% flowering, the trichome density decreased in both moisture conditions, and in this stage, the amount of thymol was observed higher in normal more than stress conditions. However, due to the decrease in trichomes density compared to the 50% flowering stage, the amount of thymol in both conditions is lower.

## Conclusions

The result of the present study showed that drought stress and harvest time influence volatile compounds of *Z. multiflora*, mainly p-cymene, γ-terpinene, myrcene, and thymol. Therefore, we can illustrate that the essential oil chemical composition is the most important quality characteristic which is affected by the harvest time. The presented results show that irrigation at 90% of the field capacity creates the best situation for the growth and production of essential oil in Z. *multiflora* because the main chemical compositions are reduced with the soil water content shortage.

The glandular trichomes density was higher at the vegetative and beginning of blooming stages than at 50 and 100% flowering stages. Peltate glandular trichomes with specialized secretory cells are the main place for the synthesis of chemical compounds. Therefore, with regards to the high density of these trichomes during vegetative and beginning of blooming stages, it is suggested that to obtain important chemical compounds with high amounts, as well as the high amount of essential oil, plant harvest is done in the two evolutionary stages mentioned above. Unlike the peltate trichomes on the leaf that were placed inside the cavity, peltate trichomes on the stem and calyx were placed on the epidermis surface. No data are available for trichomes types of *Z. multiflora* in the different development stages as a function of leaf growth. Due to the higher density of peltate glandular trichomes in normal conditions compared to stress in both 50 and 100% flowering phenological stages, the amount of main compounds especially thymol, was also observed higher in normal conditions.

Here, we provide estimates of trichomes density on leaf, stem, and calyx at four development stages during plant growth. The phonological stages of the plant have a great impact on the product yield, the amount of essential oil, and its compounds. Considering the conversion and change in the quantity and quality of essential oil compositions in different stages of plant phenology, it is possible to harvest the plant based on the need for the compounds.

## Data Availability

Data that support the findings of this study have been deposited in the Figshare.com site with this address: https://doi.org/10.6084/m9.figshare.25347673.

## Abbreviations

SEM: Scanning electron microscopy
GC: Gas chromatography
GC-MS: Gas chromatography-mass spectrometry
GC-FID-MS: Gas chromatography–flame ionization detector/mass spectrometer
HCA: Hierarchical cluster analysis
PCA: Principal component analysis
N: Normal
D: Drought stress
